# Inhibition of *Listeria monocytogenes* and *Escherichia coli* O157:H7 Growth by Ohelo Berry (*Vaccinium calycinum*) Fractions: Anthocyanins, Non-Anthocyanin Phenolics, and Organic Acids

**DOI:** 10.3390/microorganisms10112231

**Published:** 2022-11-11

**Authors:** Xiaohan Liu, Biyu Wu, Stuart T. Nakamoto, Joanne L. Imamura, Yong Li

**Affiliations:** 1 Department of Human Nutrition, Food and Animal Sciences, University of Hawaii at Manoa, 1955 East West Road, Agricultural Sciences Building 216, Honolulu, HI 96822, USA; 2 Department of Tropical Plant and Soil Sciences, University of Hawaii at Manoa, 875 Komohana Street, Hilo, HI 96720, USA

**Keywords:** ohelo berry, *Vaccinium calycinum*, anthocyanins, non-anthocyanin phenolics, organic acids, antimicrobial effect, *Escherichia coli* O157:H7, *Listeria monocytogenes*

## Abstract

*Listeria monocytogenes* and *Escherichia coli* O157:H7 are common causes of foodborne illness worldwide. Ohelo berry (*Vaccinium calycinum*) juice was found to possess inhibitory activity against *L. monocytogenes*. This study aimed to determine which constituents of ohelo berry have the most potent antimicrobial effects. The crude extract of ohelo berry was separated into sugar plus organic acids (F1), non-anthocyanin phenolics (F2), and anthocyanins (F3). The minimum inhibitory concentration (MIC) and minimum bactericidal concentration (MBC) of the fractions were determined against *L. monocytogenes* and *E. coli* O157:H7. The results demonstrated that F3 contained the highest concentrations of total phenolics and anthocyanins. All fractions caused a significant growth reduction in two bacteria compared to controls. F1 at native pH had the same MIC (1.39/0.36 Bx/acid) and MBC (5.55/1.06 Bx/acid) against the two bacteria, while neutralized F1 did not inhibit the growth of either pathogen. The MIC of F3 against *L. monocytogenes* was 13.69 mg/L cyanidin-3-glucoside equivalent, which was not affected by neutralization. Besides, *L. monocytogenes* was more sensitive than *E. coli* O157:H7 to all fractions. These findings suggest that both phenolics and organic acids contribute to the antimicrobial properties of ohelo berry, which have the potential to be used as natural food preservatives.

## 1. Introduction

Pathogens in food are one of the significant threats to human health because they may cause foodborne illnesses, ranging from gastroenteritis to some deadly complications [1]. *Escherichia coli* O157:H7 is in the top five pathogens resulting in hospitalization, whereas *Listeria monocytogenes* is among the top five pathogens leading to death [2]. Besides, massive economic losses have been caused by foodborne illness outbreaks [3]. Therefore, a variety of antibacterial measures have been proposed, and numerous antimicrobial agents have been used to prevent the infections and intoxications caused by these pathogenic bacteria [4]. Among them, natural antimicrobials have gained increasing attention since consumers prefer natural and safe food [4]. Berries, as rich resources of phytochemicals, have been extensively studied for their antimicrobial properties. Notably, cranberry extracts have been found to inhibit the growth of several pathogens, including *L. monocytogenes*, *Salmonella* spp., and *E. coli* O157:H7 [4].

*Ohelo* berry (*Vaccinium calycinum*) is a wild relative of cranberry with red berries, and is found only in Hawaii. It has been shown that *ohelo* berry juice holds antimicrobial potential against *L. monocytogenes* [5]. The inhibitory activity of ohelo berry could be attributed to its high concentrations of organic acids and polyphenolic compounds [5,6,7,8]. It is believed that the antimicrobial properties of berries relied on their low pH, but a study proved that neutralized polyphenolic extracts of cranberry still displayed inhibition against pathogens [9]. Polyphenol is a large group of organic compounds with the basic structure of aromatic ring linking hydroxyl groups (-OH). Polyphenolics have three main groups, including phenolic acids, flavonoids, and non-flavonoids [10]. Ilić et al. [11] reported that the phenolic compounds in cranberries possessed free radical scavenging properties against superoxide radical (O_2_^−^), hydrogen peroxide (H_2_O_2_), hydroxyl radicals (•OH) and oxygen (O_2_), which contributed to the inhibition of lipid peroxidation and oxidation of proteins. Anthocyanins abundantly exist in berries and belong to the flavonoid group. They are water-soluble natural pigments due to the presence of chromophores. Anthocyanins might have antioxidant, antimicrobial, anti-inflammatory, and other health-promoting properties [12]. For example, anthocyanins in blueberry show strong antibacterial effects on various foodborne pathogens [13]. 

Moreover, to better understand the health-promoting properties of berries as well as to develop new applications, in-depth studies of berry extracts are of importance. Therefore, it is necessary to classify major constituents of berry extracts and characterize their bioactive potential [14]. Since using organic solvents to extract polyphenolic compounds may introduce interfering materials, a solid-phase extraction technique has been used for the isolation and purification of polyphenolics [15]. Oszmianski and Lee [16] developed a fractionation method by using the Sep-Pak C18 cartridge to absorb bioactive compounds onto the solid phase. Sugars, organic acids, and other polar components can be eluted using acidified water, polyphenolics other than anthocyanins is then eluted by ethyl acetate, and anthocyanins can be lastly eluted using acidified methanol (0.1% HCl, *v/v*). This method has been widely used by researchers to separate bioactive compounds in berries or to isolate anthocyanins from other phenolic compounds in plants [13,17,18]. 

To our knowledge, there have been no previous studies in which bioactive compounds in ohelo berry were fractionated and investigated for their antimicrobial activities. Therefore, the objectives of this study were to (1) separate a crude extract of ohelo berry into sugar plus organic acids, non-anthocyanin phenolics, and anthocyanins, and (2) determine and compare the antimicrobial properties of these constituents against *E. coli* O157:H7 and *L. monocytogenes*.

## 2. Materials and Methods

### 2.1. Sample Preparation

Ohelo berries (*V. calycinum*) were harvested from the Island of Hawaii and stored at −80 °C. The frozen ohelo berries were freeze-dried by a freeze dryer (FreeZone, Labconco, MO, USA) and stored in a desiccator with desiccant. The desiccator was vacuumed and protected from light. Five grams of freeze-dried ohelo berries were ground into fine powder using a blender (Cuisinart, Stamford, CT, USA) and then macerated in 100 mL of methanol/water/acetic acid (80:20:0.5, *v*/*v*). The mixture was stirred overnight at 20 °C and protected from light. After vacuum filtration, collected liquid was evaporated using a rotary evaporator (IKA, Wilmington, NC, USA) under vacuum at 40 °C. After the evaporation was done, the concentrated extract was re-solubilized with distilled water to 25 mL and stored at −20 °C. 

### 2.2. Fractionation of Ohelo Berry Extract

A C18 Sep-Pak cartridge (Waters, Milford, MA, USA) was used to separate crude extract (F0) of ohelo berry into three fractions: organic acids plus sugar (F1), non-anthocyanin phenolic compounds (F2), and anthocyanins (F3) [15]. The cartridge was connected with a vacuum filter flask and vacuumed. To precondition the cartridge, 10 mL of ethyl acetate, 10 mL of absolute methanol, and 10 mL of 0.01 N aqueous HCl were passed through it sequentially. Then, 10 mL of F0 (crude extract) were loaded onto the cartridge. Thirty milliliters of acidified distilled water were used to elute organic acids and sugar as F1. Next, the cartridge was dried using a current of nitrogen gas for 10 min. After that, F2, which contained polyphenolic compounds other than anthocyanins, was eluted from the cartridge with 30 mL ethyl acetate. F3 (anthocyanins) was collected from the cartridge by passing 30 mL acidic methanol (methanol with 0.1% HCl, *v*/*v*) through it. The three fractions were then transferred into three separate round-bottom flasks. The ethyl acetate in F2 and the methanol in F3 were removed using a rotary evaporator (IKA, Wilmington, NC, USA) under vacuum at 40 °C. F1 was evaporated at 80 °C. Finally, the three concentrated fractions were collected and dissolved in 10 mL of distilled water. The three fractions were stored at −20 °C for no longer than two weeks. Fractions were filter-sterilized before performing all the experiments for analyzing their antimicrobial properties.

### 2.3. Chemical Analysis of Ohelo Fractions

To determine the chemical characteristics of the crude extract and each fraction, five assays were performed. A pH meter was used to measure the pH of each sample (Oaklon, OH, USA). The crude extract and three fractions were individually measured for the ratio of °Bx to titratable acidity [19]. Soluble sugar solids in each sample were quantified using a refractometer (Bio-Rad, iMark, Hercules, CA, USA). Each sample was titrated with 0.1 M NaOH to determine the concentration of organic acids in citric acid equivalents. The concentrations of total phenolic contents of the crude extract and three fractions were measured by the Folin–Ciocalteu method and reported as grams of gallic acid equivalents (GAE) per milliliter of extracts [15]. Each sample was measured for the concentration of anthocyanins using the pH differential method. This method relies on the structural change of the anthocyanin chromophore at pH 1.0 and pH 4.5 [20]. The results were reported in equivalents of cyanidin-3-glucoside. 

### 2.4. Bacterial Strains and Growth Conditions

Two pathogenic bacteria, *E. coli* O157:H7 C7927 and *L. monocytogenes* F2365 were used in this study. The strains were cultured and purified on MacConkey Sorbitol Agar (MSA, Difco^TM^, Sparks, MD, USA) and Oxford Medium Agar (MOX, Difco^TM^, Sparks, MD, USA), respectively. The strains were grown in Tryptic soy broth (TSB, BBL^TM^, Sparks, MD, USA) at 37 °C for 24 h before being used in the following experiments of antimicrobial activities.

### 2.5. Agar Well Diffusion Assay

The cultures of *E. coli* O157:H7 and *L. monocytogenes* were individually diluted to 5 log CFU/mL using 0.1% peptone water. One milliliter of diluted bacterial solution and 15 mL of melted Mueller-Hilton agar (Difco^TM^, USA) were mixed in a Petri dish. After the agar was solidified, wells were created using a sterilized glass Pasteur pipette (ASTMTM E 732 Fisherbrand, Chino, CA, USA). 50 uL volumes of crude extract, each fraction, 10% (*v*/*v*) bleach (positive control), and sterilized distilled water (negative control) were loaded into the wells. The plates were incubated at 37 °C for 24 h, and the inhibition zones were measured using a ruler.

### 2.6. Determination of the Minimum Inhibitory Concentration (MIC) and Minimum Bactericidal Concentrations (MBC)

The MIC and MBC of each fraction were determined by broth dilution method according to Clinical and Laboratory Standards Institute (CLSI) guidelines (document M07) [21]. *E. coli* O157:H7 and *L. monocytogenes* were diluted to 6 log CFU/mL with 2× Mueller Hinton (MH) broth. Each extract was prepared as 0-fold, 2-fold, 4-fold, 8-fold, 16-fold, 32-fold, and 64-fold dilutions with sterilized water. After that, each fraction dilution and 6 log CFU/mL of bacterial solution were mixed in a sterilized microcentrifuge tube with 2× MH broth to yield final extract concentrations of 50%, 25%, 12.5%, 6.25%, 3.125%, 1.56%, and 0.78% and bacterial concentration of 5 log CFU/mL. The positive control was MH broth with 5 log CFU/mL of bacteria. A negative control was uninoculated MH broth. Samples were incubated at 37 °C for 24 h. Viable cell counts of each sample were determined at both 0 and 24 h on Plate Count Agar (PCA). MIC was established as the lowest concentration of extract that inhibited the visible growth of tested bacteria after overnight incubation. MBC was determined as the lowest concentration of the extract that killed tested bacteria. MIC and MBC were determined by comparing the bacterial counts of controls and treatments at 0 and 24 h [22].

### 2.7. Statistical Analysis

All tests were performed three times. The inhibition zone diameters from different treatments were compared. The viable cell counts of tested bacteria were converted to log CFU/mL. The counts of treatments at 24 h were compared with treatments at 0 h and the control at 24 h. The data were analyzed via analysis of variance (ANOVA) with a significance level of 0.05 utilizing Statistical Package for the Social Sciences (SPSS 24.0, IBM Corp., Armonk, NY, USA). 

## 3. Results

### 3.1. Characterization of Ohelo Berry Fractions

Crude extract (F0) of ohelo berry was separated by a C-18 Sep-Pak cartridge into three fractions. Fraction 1 (F1) contained most of the sugar plus organic acids, fraction 2 (F2) contained the polyphenolics other than anthocyanin, and fraction 3 (F3) was the vast majority of anthocyanins. The chemical characteristics of all the fractions are shown in Table 1. All three fractions showed high acidity with a pH of about 2 or 3. The data demonstrated that after separation, F1 almost did not contain any phenolics or anthocyanins. Among all the three fractions, F3 contained the highest concentrations of total phenolics and anthocyanins, with 9.28 mg/mL gallic acid equivalent and 438.18 mg/L cyanidin-3-glucoside equivalent, respectively. 

### 3.2. Inhibition Zones

**Figure 1:**
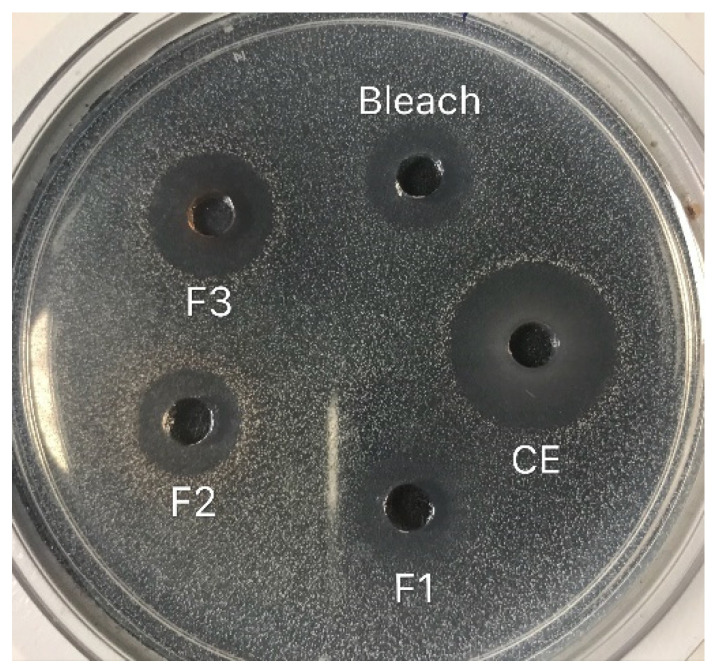
Typical inhibition zones (mm) generated by native ohelo berry crude extract and fractions against *L. monocytogenes*. Bleach: 10% (*v*/*v*) bleach; CE: ohelo berry crude extract (F0); F1: sugar plus organic acids; F2: non-anthocyanin phenolics; F3: anthocyanins.

The sizes of inhibition zones generated by ohelo berry crude extract and fractions against *E. coli* O157:H7 and *L. monocytogenes* are recorded in Table 2. Figure 1 shows typical inhibition zones generated by native ohelo berry crude extract and fractions against *L. monocytogenes.* Both clear and unclear zones were generated against Gram-negative *E. coli* O157:H7. The crude extract of ohelo berry was the most potent antimicrobial with the larger inhibition zones against both tested pathogens than three fractions, suggesting the antimicrobial activities of ohelo berry were contributed by a combination of all the bioactive compounds. Among the three fractions, *E. coli* O157:H7 was more sensitive to F1, while the largest inhibition zone (*p* < 0.05) against *L. monocytogenes* was generated by F3, which was 14.25 mm. After neutralization, the antimicrobial effects of the crude extract and three fractions were not as strong as at native pH. In particular, neutralized F1 showed no inhibition effects on either pathogenic bacteria. However, neutralized crude extract still generated comparable inhibition zones against both tested pathogens to 10% bleach. F2 and F3 generated no inhibition zone against *E. coli* O157:H7 after neutralization. Neutralized F2 and F3 yielded significantly smaller zones against *L. monocytogenes* than they did at native pH.

### 3.3. MIC and MBC

**Figure 2:**
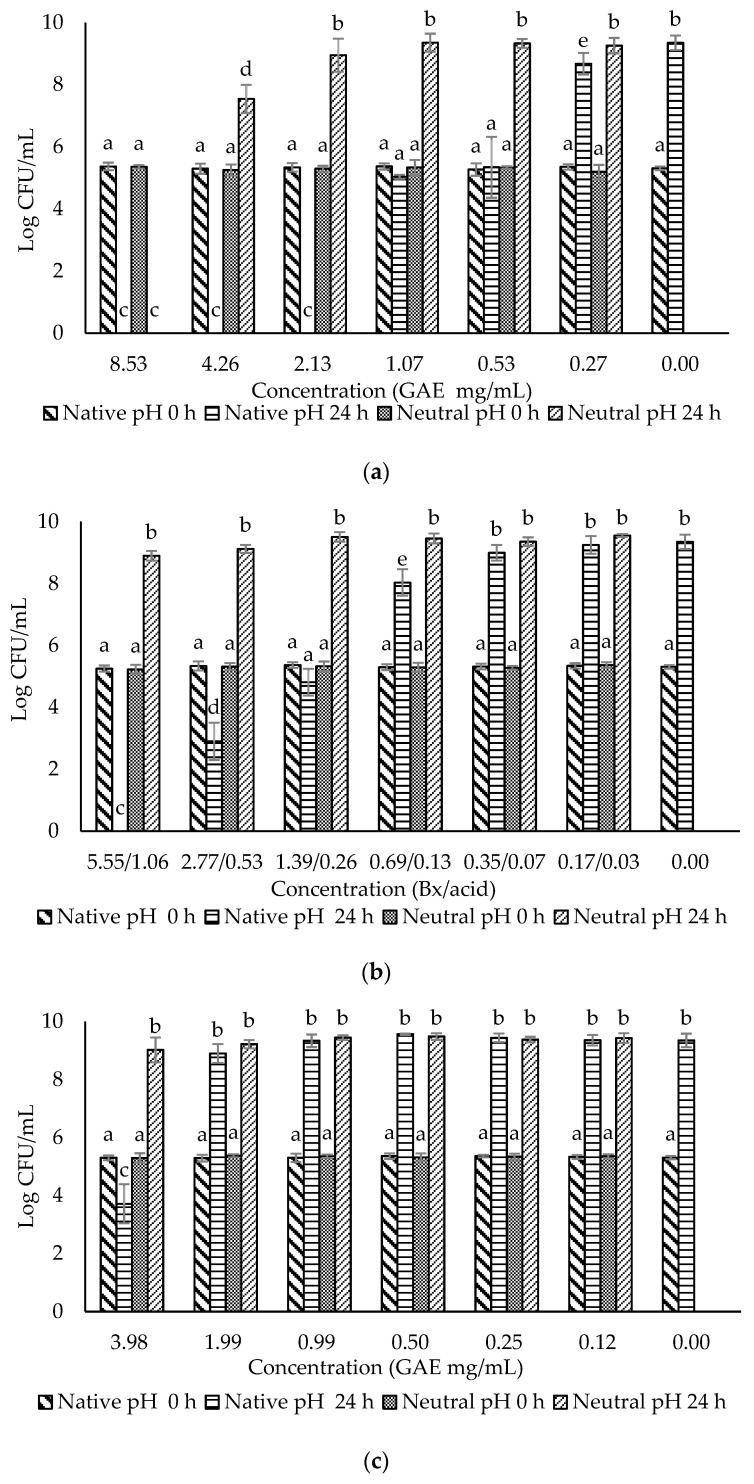

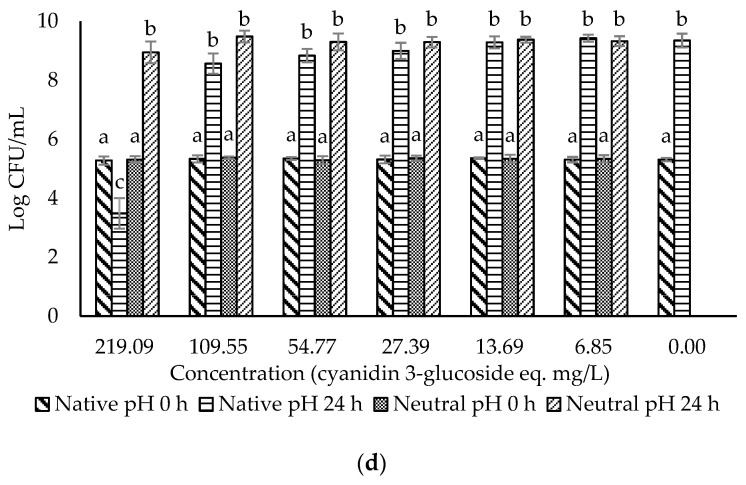
Evaluation of the antimicrobial effects of (**a**) F0 (ohelo berry crude extract); (**b**) F1 (sugar plus organic acids); (**c**) F2 (non-anthocyanin phenolics); (**d**) F3 (anthocyanins) against *Escherichia coli* O157:H7. The viable cell counts were determined at 0 h and 24 h. Means with different superscript letters a to e are significantly different at *p* < 0.05.

**Figure 3:**
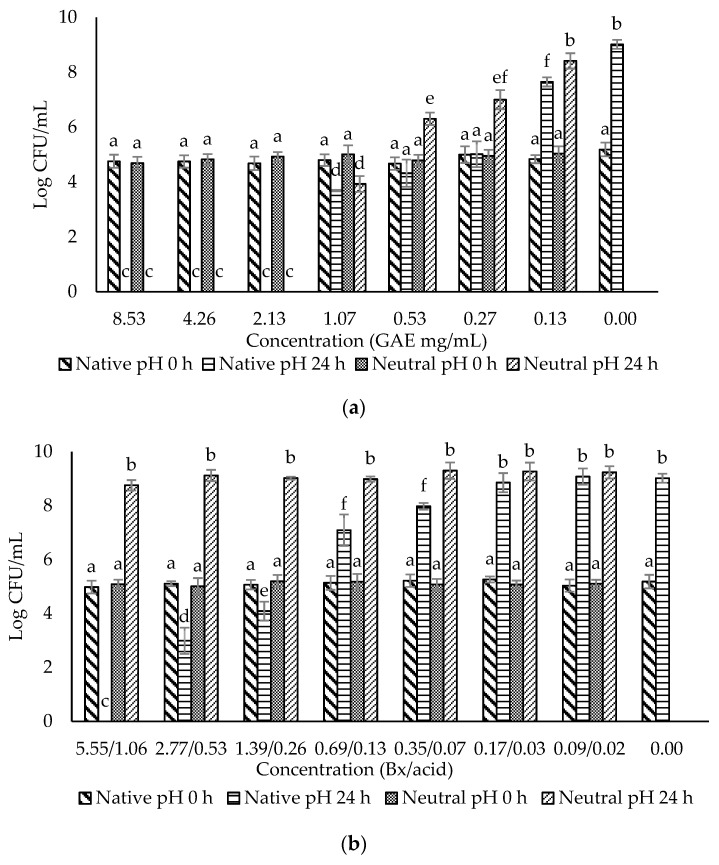

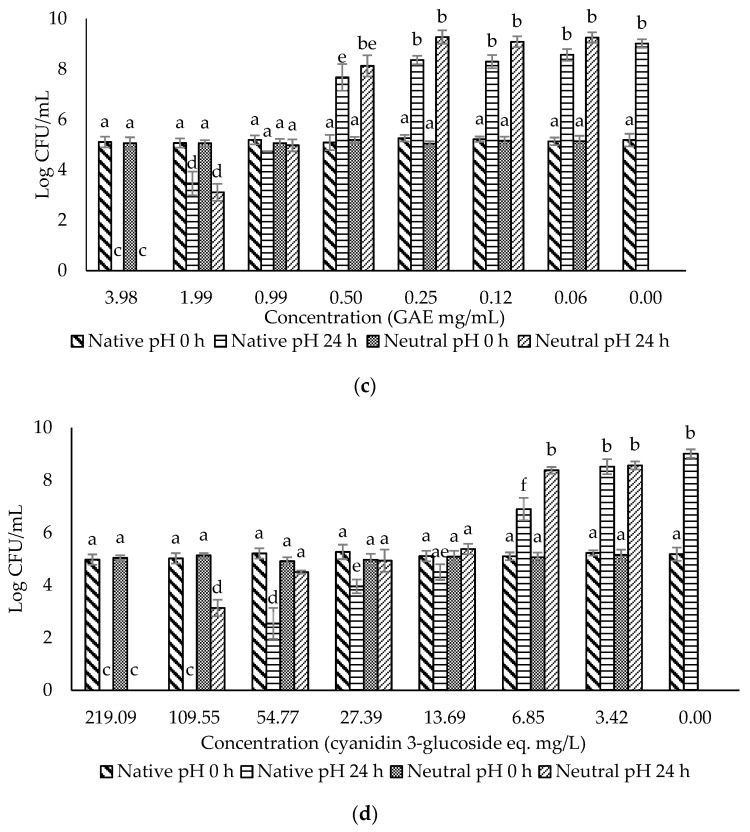
Evaluation of the antimicrobial effects of (**a**) F0 (ohelo berry crude extract); (**b**) F1 (sugar plus organic acids); (**c**) F2 (non-anthocyanin phenolics); (**d**) F3 (anthocyanins) against *Listeria monocytogenes*. The viable cell counts were determined at 0 h and 24 h. Means with different superscript letters a to f are significantly different at *p* < 0.05.

The crude extract and three fractions of ohelo berry were neutralized using NaOH, and their MIC and MBC at both native pH and neutral pH were determined against *E. coli* O157:H7 and *L. monocytogenes*. The treatment pH of diluted crude extract and fractions of ohelo berry are presented in Table 3. The viable cell counts were determined for each pathogen treated by the crude extract and three fractions at both native pH and neutralized pH. The results for *E. coli* O157:H7 and *L. monocytogenes* are presented in Figure 2 and Figure 3, respectively. Then, a summary of MIC and MBC are shown in Table 4. Although the three fractions showed weaker antibacterial effects than the crude extract, fractions at native pH produced a significant reduction (*p* < 0.05) of all the bacteria compared with the control at 24 h. F1 at native pH had similar MIC (1.39/0.36 °Brix/acid) and MBC (5.55/1.06 °Brix/acid) against the two bacteria. However, F2 and F3 at native pH had stronger antimicrobial effects on Gram-positive *L. monocytogenes* than Gram-negative *E. coli* O157:H7. Diluted F2 and F3 had neutral pH when their concentrations were lower than 0.25 mg mL^−1^ GAE and 27.39 eq. mg mL^−1^ C3G, respectively, which showed inhibition effects against *L. monocytogenes*. It was evident that F1 lost the antimicrobial activity against both bacteria after neutralization. These results were in agreement with the previous results of inhibition zones experiments. However, the MIC and MBC of F2 and F3 against *L. monocytogenes* were not affected considerably by neutralization. Neutralized crude extract of ohelo berry still showed antimicrobial effects on both tested pathogens.

## 4. Discussion

Cranberry has been utilized as a functional fruit due to its high contents of bioactive compounds [23], which were found to have antimicrobial activities [9,17,24]. Hummer et al. [25] reported ohelo berry (*Vaccinium calycinum*) has a higher level of phenolics than cranberry. Our previous study revealed that the ohelo berry juice exhibited promising antimicrobial efficacy against *L. monocytogenes* in skim and whole milk [5]. The present study was the first to determine which constituents of ohelo berry have the most potent antimicrobial properties against pathogenic bacteria. The bioactive compounds of ohelo berry extract were divided into three fractions, including sugar plus organic acids, non-anthocyanin phenolics, and anthocyanins by a separation method using the Sep-Pak C18 cartridge, which was initially developed by Oszmianski and Lee [16]. 

All the fractions were analyzed for Brix, titratable acidity, total phenolics, and anthocyanin contents with the crude extract used as reference. The results of chemical analysis illustrated the Sep-Pak C18 cartridge could effectively separate the constituents in ohelo berry. Additionally, the highest concentrations of total phenolics and anthocyanins were observed in the fraction of anthocyanins among all three fractions. The antimicrobial effects of crude extract and all the fractions at both native pH and neutral pH on *E. coli* O157:H7 and *L. monocytogenes* were analyzed in this study. Sugar plus organic acids had similar antibacterial effects on these two bacteria with the same MIC and MBC. However, *L. monocytogenes* was more sensitive to phenolics and anthocyanins than *E. coli* O157:H7, as shown by larger inhibition zones and lower MIC and MBC. Similar results were observed by Lau et al. who indicated that *L. monocytogenes* was more sensitive to cranberry extract than *E. coli* O157:H7 and *S*. Enteritidis [26]. The hypothesis was that organic acids could be more effective than phenolics and anthocyanins in destroying the outer membranes of Gram-negative bacteria, which is in agreement with the results of Lacombe et al. [17], who used a transmission electron microscope (TEM) to visualize the damage of *E. coli* O157:H7 cells treated by three fractions of cranberry. Their TEM images displayed the lack of distinguishable outer membrane in the cells treated with sugar plus organic acids, while localized disintegration of the outer membrane was observed in the cells treated with phenolics and anthocyanins. In a previous report, the quinic and citric acids were the dominant organic acids in ohelo berry, followed by malic and shikimic acid [25]. Citric acid, naturally occurring in citrus fruits, has been extensively reported for its growth inhibition effects on pathogenic bacteria [27]. Quinic acid also exhibited inhibitory effects on biofilm formation of *Pseudomonas aeruginosa* [28]. Malic acid present in blackberries and cherries has shown strong antimicrobial properties for preventing the growth of *L. monocytogenes* and *E. coli* O157:H7 [29]. It is suggested that the low pH in berries contributed to their antimicrobial activity. Eswaranandam et al. [29] revealed that the MIC of citric acid against *Salmonella* Typhimurium increased from 0.312% (*v/v*) to 0.625% (*v/v*) after neutralization. Moreover, our study revealed that sugar plus organic acids of ohelo berry had no antimicrobial activity after they were neutralized, but neutralized phenolics and anthocyanins of ohelo berry still had strong antimicrobial effects on tested pathogens (Table 4). This finding is important since the application of ohelo berry products as antimicrobials would not be limited to the acidity of ohelo berry. 

Polyphenols are widespread in various plants, which greatly contribute to the biological effects of plants and plant-derived materials. Regarding our results, both polyphenolics and organic acids contribute to the antimicrobial properties of ohelo berry. The organic acids could be protonated and cross both the cell membranes of Gram-positive bacteria and the outer membranes and cell membranes of Gram-negative bacteria into the bacterial cytoplasm. Then, the growth of bacteria was inhibited because adenosine 5′-triphosphate (ATP) had to be used to pump the protons out of the cell [8]. Besides the high concentration of hydrogen ions, the polyphenolic compounds also contribute to the inhibition of pathogens by disrupting cell membranes, inactivating enzymes, and other possible mechanisms [30]. For Gram-negative bacteria, the outer membrane is an effective barrier to block off hydrophilic compounds [7]. Gram-positive bacterial cells were more sensitive to berry phenolics while they were protected by the thick peptidoglycan cell walls. However, the polyphenolic compounds in berries were found to have membrane interaction activities, which can release lipopolysaccharide (LPS) and increase the permeability of outer membranes of Gram-negative bacteria [7]. Additionally, polyphenolics could sequester free iron which is essential for the survival and virulence of bacteria [31]. Moreover, He et al. [32] suggested that flavonoids could interact with hydrophilic regions on the cell membrane, followed by permeating into the hydrophobic region when the concentration of flavonoids increased. Then, the bacteria might be inhibited via changes of the liposome fluidity. Chlorogenic acid was reported as a dominant phenolic acid in ohelo berry [25]. Studies demonstrated that chlorogenic acid could disrupt the cell membrane, causing the damage to cell permeability and resulting in the leakage of intracellular materials [33]. In addition, anthocyanins are an important type of flavonoid and have significant antibacterial effects on foodborne pathogens. Anthocyanins could have profound impacts on pathogenic bacteria by damaging their cell membranes and affecting related enzymes [13]. Besides, the tricarboxylic acid (TCA) cycle and the biosynthesis of bacterial cells could be decreased by anthocyanins [13]. However, the exact modes of action for these bioactive compounds remain unclear. Therefore, the antimicrobial activities of ohelo berry rely on a synergistic effect of all the bioactive compounds, making it complex to clarify the antimicrobial mechanisms of these functional components in the mixtures. 

Anthocyanins (F3) could be further separated into anthocyanins and proanthocyanidins. The proanthocyanidins in cranberry were found to play an essential role in increasing the permeability of outer membranes and destroying the lipopolysaccharide structures of Gram-negative bacteria [24,34]. In addition, the proanthocyanidins extracted from grape seed could inhibit bacterial adhesion and coaggregation, and reduce biofilm formation of *P. aeruginosa* [35]. Hence, the proanthocyanidins in ohelo berry should be further studied since Hummer et al. [25] claimed that the proanthocyanidin content of ohelo berry was higher than cranberry. 

In addition, since organic acids and phenolic compounds in fruit juice still showed strong antibacterial activities after pasteurization [36], ohelo berry juice could be commercially produced as a functional food. Overall, ohelo berry has the potential to be used as natural antibacterial agents in the food industry. Future work on its inhibition of pathogenic bacteria in the host is needed. 

It is generally believed that the antimicrobial activity of berries relies on their low pH. However, the neutralized crude extract of ohelo berry still showed the inhibitory and bactericidal effects on *E. coli* O157:H7 and *L. monocytogenes*. Moreover, after ohelo berry extract was separated into three fractions, not only the organic acids fraction but also the non-anthocyanin phenolics and anthocyanins fractions inhibited the growth of the tested pathogens. Further, the antimicrobial activities of those phenolic compounds were almost not affected by neutralization. Thereby, the polyphenolic compounds of ohelo berry have the potential as food preservatives, and their application would not be limited to acidic foods. 

## 5. Conclusions

There is an increasing interest in natural antimicrobials as alternative treatment strategies in bacterial infections. Ohelo berry is a native Hawaiian plant. To our knowledge, the present study is the first report demonstrating the antimicrobial potential of ohelo berry fractions against pathogenic bacteria. *L. monocytogenes* was found to be more sensitive to phenolics and anthocyanins than *E. coli* O157:H7. The antimicrobial effects of ohelo berry phenolics and anthocyanins on *L. monocytogenes* were not influenced by neutralization. However, neutralized ohelo berry phenolics and anthocyanins could not inhibit the growth of *E. coli* O157:H7 at the test concentrations. To sum up, ohelo berry has the potential as a functional fruit and a natural antimicrobial for preserving food. This study laid the foundation for further investigating the antimicrobial mechanisms of ohelo berry fractions and exploring the potential utilization of ohelo berry for human health.

## Figures and Tables

**Table 1:** The pH, °Bx, titratable acidity, the concentration of total phenolics, the concentration of anthocyanins of ohelo berry extract and fractions ^†^.

Fraction *	pH	Sugar and Organic Acids (°Bx/Acid)	Total Phenolics (Gallic Acid Equivalent, mg/mL)	Anthocyanins (cyanidin-3-glucoside Equivalents, mg/L)
F0	3.14 ± 0.06 ^a^	16.3 ± 0.4 ^a^/2.88 ± 0.15 ^a^	17.05 ± 0.04 ^a^	462.73 ± 11.52 ^a^
F1	2.68 ± 0.09 ^b^	15.3 ± 0.6 ^a^/2.13 ± 0.19 ^b^	0 ^d^	0 ^c^
F2	2.51 ± 0.03 ^b^	0 ^c^	7.95 ± 0.01 ^b^	20.54 ± 0.83 ^b^
F3	2.05 ± 0.05 ^c^	0 ^c^	9.28 ± 0.30 ^c^	438.18 ± 2.00 ^a^

^†^ Means in the same column with different superscript letters a to d are significantly different at *p* < 0.05. * F0: ohelo berry crude extract; F1: Sugar plus organic acids; F2: non-anthocyanin phenolics; F3: anthocyanins.

**Table 2:** The inhibition zones (mm) generated by ohelo berry crude extracts and fractions against *E. coli* O157:H7 and *L. monocytogenes* ^†^.

Treatment		Bacteria	
		*Escherichia coli* O157:H7	*Listeria monocytogenes*
Native pH	F0	10 ± 0.25 ^b^/20.5 ± 0.5 * ^A^	20.75 ± 0.25 ^a^
	F1	- ^‡^/13 ± 1 * ^C^	12 ± 1.5 ^c^
	F2	-/9.25 ± 0.25 * ^D^	9.75 ± 0.25 ^d^
	F3	-/11.5 ± 0.5 * ^CD^	14.25 ± 0.25 ^b^
Neutralized	F0	9.25 ± 0.25 ^b^/11 ± 0 * ^CD^	13 ± 0 ^b^
	F1	-	-
	F2	-	8 ± 0 ^e^
	F3	-	11.25 ± 0.25 ^c^
Bleach (10% *v v*^−1^)		13 ± 0 ^a^/16.5 ± 0.5 * ^B^	13.25 ± 0.25 ^b^

^†^ F0: Ohelo berry crude extract; F1: Sugar plus organic acids; F2: non-anthocyanin phenolics; F3: anthocyanins. * The diameter of unclear inhibition zone generated by the treatment against Gram-negative bacteria. Means in the same column with different superscript letters a to e are significantly different at *p* < 0.05 for the size of the clear inhibition zone. Means in the same column with different superscript letters A to D are significantly different at *p* < 0.05 for the size of the unclear inhibition zone. ^‡^ No inhibition zone was observed.

**Table 3:** The pH value of diluted ohelo berry extract and fractions.

Fraction ^†^	50%	25%	12.5%	6.25%	3.12%	1.56%	0.78%
F0	3.48 ± 0.05	3.78 ± 0.03	4.15 ± 0.05	4.46 ± 0.04	4.89 ± 0.02	5.72 ± 0.03	6.57 ± 0.03
F1	3.12 ± 0.02	3.58 ± 0.02	4.09 ± 0.06	4.80 ± 0.04	5.93 ± 0.07	6.48 ± 0.03	6.73 ± 0.05
F2	3.89 ± 0.01	4.39 ± 0.04	5.21 ± 0.05	6.18 ± 0.05	6.57 ± 0.03	6.74 ± 0.04	6.84 ± 0.02
F3	4.46 ± 0.03	5.58 ± 0.03	6.34 ± 0.06	6.64 ± 0.03	6.79 ± 0.02	6.87 ± 0.05	6.91 ± 0.03

^†^ F0: ohelo berry crude extract; F1: Sugar plus organic acids; F2: non-anthocyanin phenolics; F3: anthocyanins.

**Table 4:** Minimum inhibitory concentration (MIC) and minimum bactericidal concentrations (MBC) of ohelo berry extract and fractions against *E. coli* O157:H7 and *L. monocytogenes* ^†^.

Fraction		*E. coli* O157:H7		*L. monocytogenes*	
		MIC	MBC	MIC	MBC
Native pH	F0 (GAE, mg mL^−1^)	0.53	2.13	0.27	2.13
	F1 (Bx/acid)	1.39/0.26	5.55/1.06	0.69–1.39/ 0.13–0.26	5.55/1.06
	F2 (GAE, mg mL^−1^)	1.99–3.98	NA *	0.99	3.98
	F3 (C3G eq., mg L^−1^)	109.55–219.09	NA	6.85–13.69	109.55
Neutralized	F0 (GAE, mg mL^−1^)	4.26–8.53	8.53	0.53–1.07	2.13
	F1 (Bx/acid)	NA	NA	NA	NA
	F2 (GAE, mg mL^−1^)	NA	NA	0.99	3.98
	F3 (C3G eq., mg L^−1^)	NA	NA	13.69	219.09

^†^ F0: Ohelo berry crude extract; F1: Sugar plus organic acids; F2: non-anthocyanin phenolics; F3: anthocyanins. * Not applicable.

## Data Availability

Not applicable.
